# Toward targeted therapy in chemotherapy-resistant pancreatic cancer with a smart triptolide nanomedicine

**DOI:** 10.18632/oncotarget.7073

**Published:** 2016-01-29

**Authors:** Cheng Wang, Biao Liu, Xuelian Xu, Bo Zhuang, Hongxia Li, Jiaqi Yin, Mengyi Cong, Wei Xu, Aiping Lu

**Affiliations:** ^1^ Key Laboratory of Marine Drugs, Chinese Ministry of Education, School of Medicine and Pharmacy, Ocean University of China, Qingdao, China; ^2^ Institute for Advancing Translational Medicine in Bone and Joint Diseases, Jockey Club School of Chinese Medicine Building, Hong Kong Baptist University, Kowloon Tong, Kowloon, Hong Kong; ^3^ Department of Pharmacy, Shandong Provincial Qian Foshan Hospital, Jinan, China; ^4^ Division of Molecular Pharmaceutics and Center for Nanotechnology in Drug Delivery, Eshelman School of Pharmacy, University of North Carolina at Chapel Hill, Chapel Hill, NC, USA

**Keywords:** chemoresistance, pancreatic cancer, triptolide, AS1411, PEG-PDLLA

## Abstract

Chemoresistance is the major impediment for treating pancreatic cancer. Herb-derived compound triptolide (TP) can inhibit proliferation of chemo-resistant pancreatic cancer (CPC) cell lines through multiple mechanisms, which exhibited superior anticancer efficacy compared with gemcitabine. However, toxicity due to non-specific exposure to healthy tissues hindered its clinical translation. Herein we successfully achieved targeting CPC cells and avoiding exposure to healthy tissues for TP by nucleolin-specific aptamer (AS1411) mediated polymeric nanocarrier. We conjugated AS1411 aptamer to carboxy terminated poly(ethylene glycol)–block–poly(d, l-lactide) (HOOC-PEG-PDLLA), then prepared AS1411-PEG-PDLLA micelle loading TP (AS-PPT) through solid dispersion technique. AS-PPT showed more antitumor activity than TP and equivalent specific binding ability with gemcitabine-resistant human pancreatic cancer cell (MIA PaCa-2) to AS1411 aptamer *in vitro*. Furthermore, we studied the distribution of AS-PPT (Cy3-labed TP) at tissue and cellular levels using biophotonic imaging technology. The results showed AS1411 facilitated TP selectively accumulating in tumor tissues and targeting CPC cells. The lifetime of the MIA PaCa-2 cell-bearing mice administrated with AS-PPT was efficiently prolonged than that of the mice subjected to the clinical anticancer drug Gemzar^®^ *in vivo*. Such work provides a new strategy for overcoming the drug resistance of pancreatic cancer.

## INTRODUCTION

Pancreatic cancer (PC) is one of the most aggressive human malignancies with a historically dismal long-term prognosis, which accounts for 2% of all cancers but 6% of cancer deaths worldwide [1]. Chemoresistance is the major impediment for treating PC, which can arise de novo or can be acquired (therapy-induced) refractoriness to anticancer chemotherapeutic interventions [2–5]. Gemcitabine has been recognized by many oncologists as the first-line drug to treat pancreatic cancer, however, insensitivity due to singleness of therapeutic target is the major concern during its clinical application [6–9]. Hence, developing novel chemotherapeutic drug to overcome the drug resistance of pancreatic cancer is becoming a real challenge.

**Figure 1:**
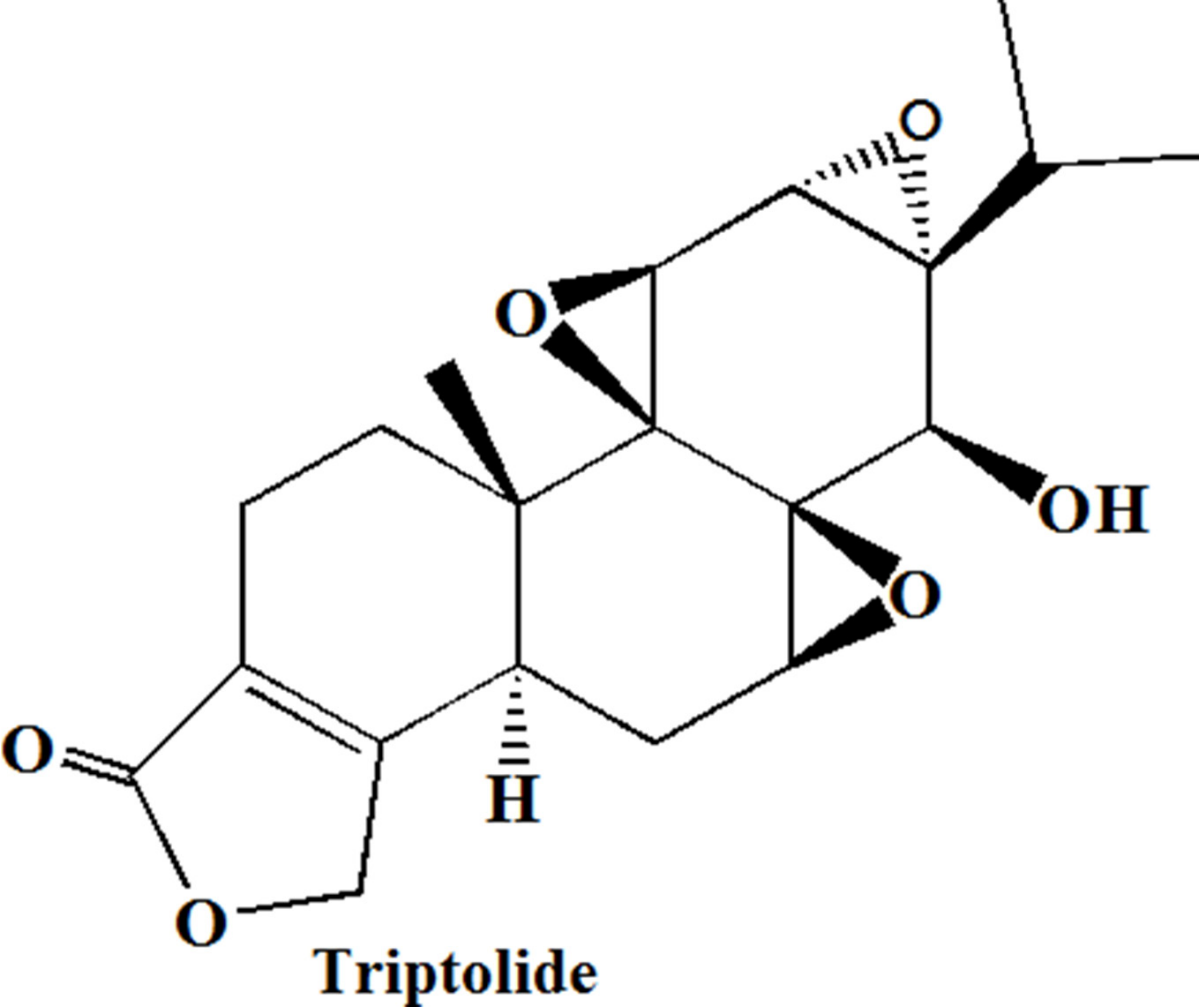
Chemical Structures of triptolide

Chemoresistance in PC is triggered by various molecular mechanisms including aberrant gene expression, mutations, deregulation of key signaling pathways (such as NF-κB, Akt, and apoptosis pathways), epithelial-mesenchymal transition (EMT) and the presence of stroma cells, highly resistant cells and stem cells [2–5]. Each of those mechanisms contributes to drug resistance in pancreatic cancer from different aspects, and recommends different therapeutic targets. Triptolide (TP, Figure 1 is the structure of Triptolide) is a major active compound of *Tripterygium wilfordii Hook F*, which can inhibit proliferation of chemo-resistant pancreatic cancer cells (CPC) through multiple pathways, such as inhibition of P-glycoprotein, Heat Shock Protein 70 (HSP70), NF-κB, Bax gene and angiogenesis, etc, which exhibited superior anticancer efficacy compared to gemcitabine and taxanes *in vitro* [6, 10–17]. However, TP-induced toxicity due to non-specific exposure to healthy tissues (e.g. liver) hindered its clinical translation. In addition, higher intratumoral accumulation is difficult for anticancer agents with no specific ligands, of note, one major mechanism of DR is the efflux of drug from the cell by P-glycoprotein, thereby keeping the intracellular levels below the killing threshold [18]. Thus, realizing TP selectively targeting PC tissues or cells to enhance uptake and simultaneouly reduce side effect may be practical to reverse DR with longer survival time.

Nucleolin overexpressed on the plasma membrane of PC cells compared with normal cells, which can work as a cell surface receptor [19, 20]. Aptamers are single-stranded oligonucleotides which can specifically recognize and bind to targets by distinct secondary and tertiary structures. Aptamer, as target moieties, conjugated to nano-vehicles have been studied in many research articles [21–24]. Nucleolin-specific aptamer (AS1411, 5′GGTGGTGGTGGTTGTGGTGGTGGTG G-3) is a G-rich phosphodiester oligonucleotide with high affinity and specificity to nucleolin, which has been proved to be safe in clinical research [25]. Then, this provide us an inspiration of developing AS1411-mediated nanoparticle to achieve CPC-specific delivery of TP.

**Figure 2:**
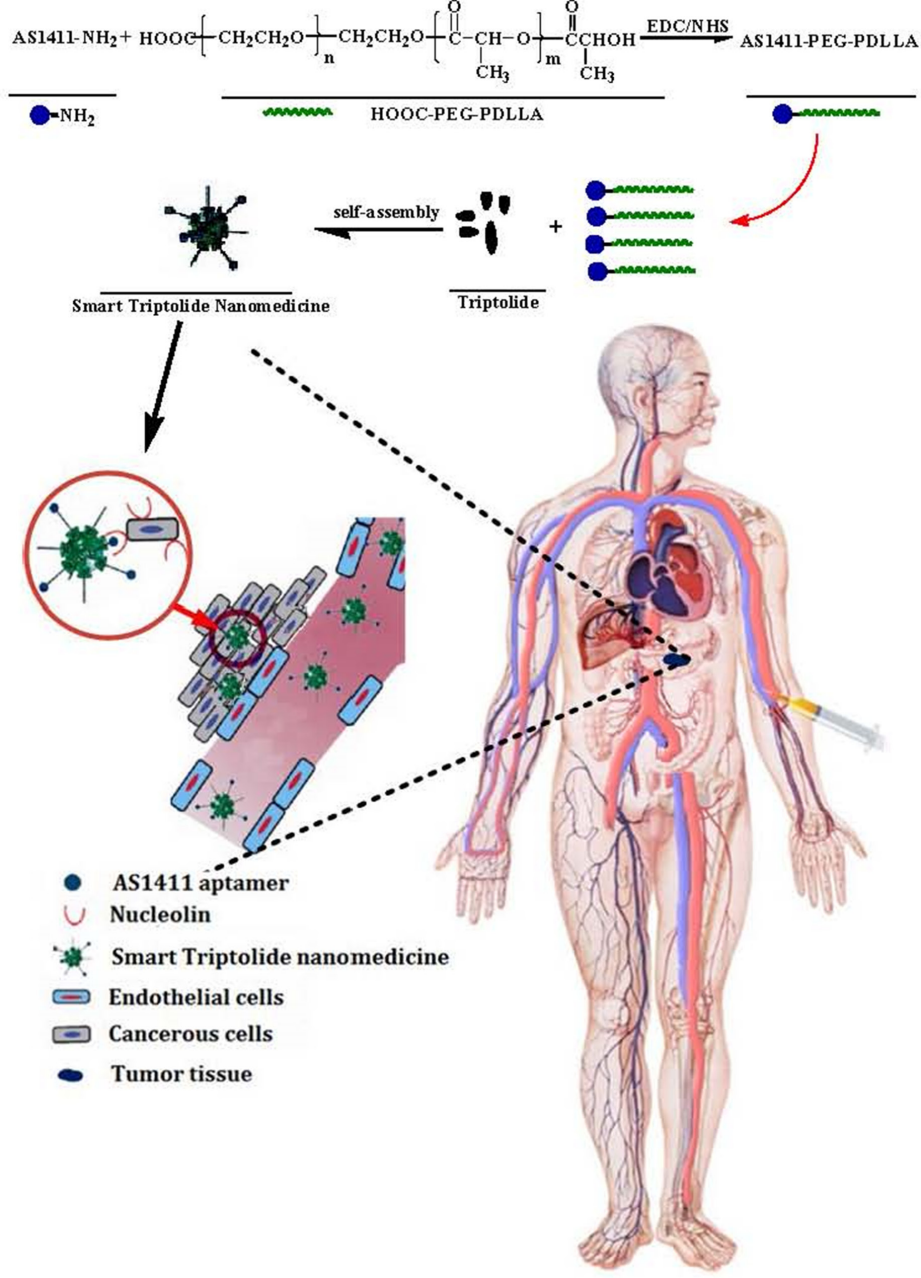
Schematic representation of synthesizing AS1411-PEG-PDLLA and preparing smart triptolide nanomedicine, which selectively targeting CPC cells

In pilot study, we have developed triptolide-polymeric micelle (TP-PM) by polymer-based delivery system, which not only overcame the pitfall of hydrophobicity, but also facilitated TP passively accumulating in cancerous tissues based on the Enhanced Permeability and Retention (EPR) effect [26]. Nevertheless, as passive targeting nanoscale preparations, TP-PM suffers from some limitations including the degree of tumor vascularization and angiogenesis, high interstitial fluid pressure of solid tumors leading to drug expulsion and opsonins interaction, etc [27, 28], which limited its therapeutic effect. Herein, we conjugated AS1411 to TP micelle composed with poly(ethylene glycol)–block–poly (D, L-lactide) (PEG-PDLLA) diblock copolymers, which was termed AS-PPT (Figure 2). PEG-PDLLA was chosen as a drug carrier for TP due to its biodegradability and biocompatibility properties, which has been applied in the formulation of Genexol-PM^®^ for treatment of metastatic carcinoma of ovary and breast after failure of first-line or subsequent chemotherapy [29, 30]. In this study, we investigated the tissue and cellular levels distribution of AS-PPT (Cy3-labed TP) using biophotonic imaging. The results showed AS1411 could facilitate TP selectively targeting CPC cells and accumulating in tumor tissues. In addition, AS-PPT possess more antitumor efficacy than nontargeted TP-PM in a xenograft mouse model of gemcitabin-resistant human pancreatic cancer (MIA PaCa-2), and the lifetime of the MIA PaCa-2 cell-bearing mice administrated with AS-PPT was efficiently prolonged than that of the mice subjected to the clinical anticancer drug Gemzar^®^ *in vivo*. These indicated AS1411 aptamer can facilitate TP selectively targeting CPC cells and accumulating in tumor tissues, AS-PPT may be a potential active targeting drug for CPC.

## RESULTS AND DISCUSSION

### Characterization of AS1411-PEG-PDLLA polymer and AS-PPT

**Figure 3:**
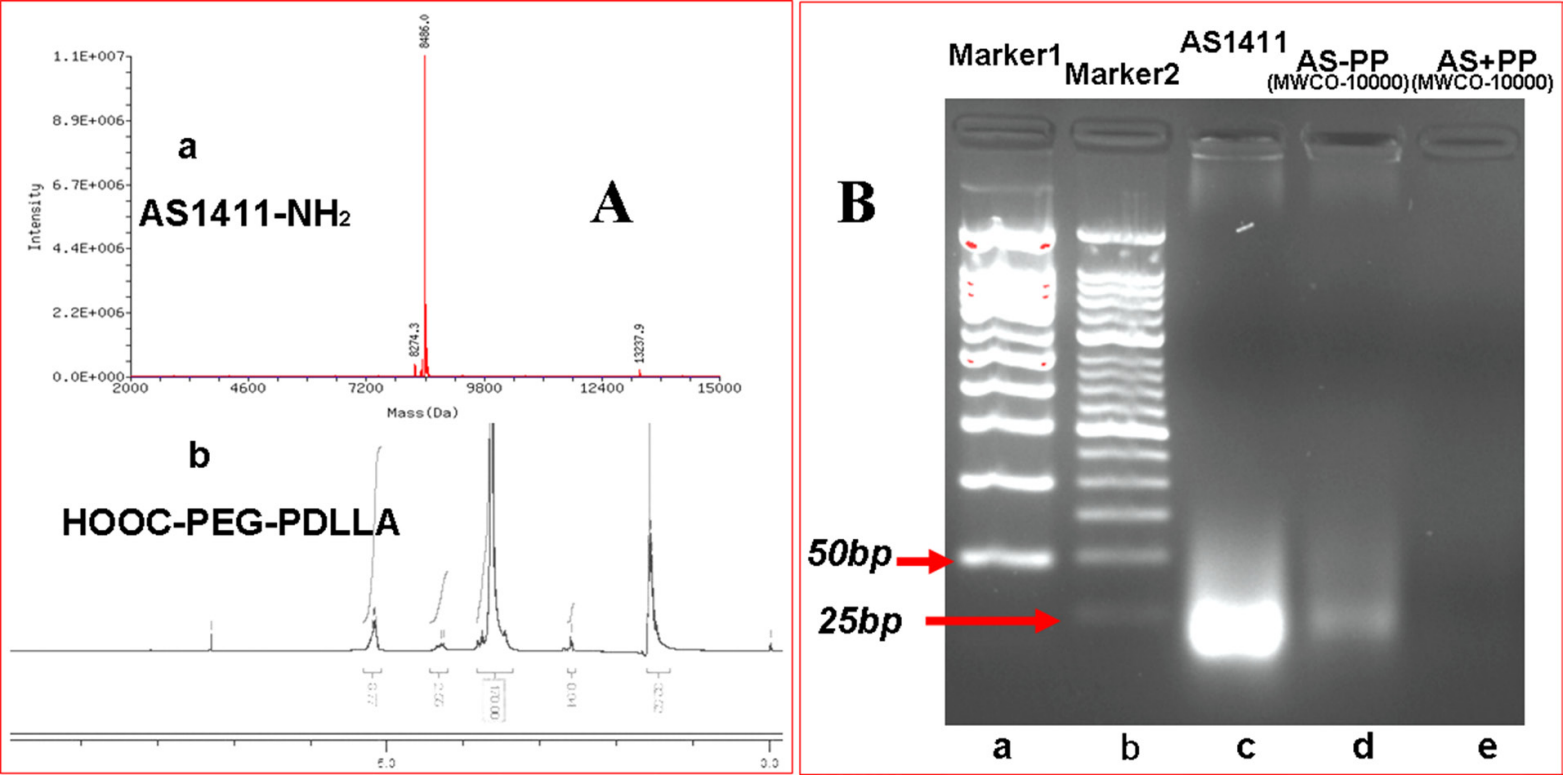
Characterization of A (**a**). The MS spectrum of Nucleolin Aptamer (AS1411-NH_2_). A(b). The 1H NMR spectrum of HOOC-PEG-PDLLA polymeric material. **Characterization of B** (a). Marker1 50 bp, B (**b**). Marker2 25 bp, B (**c**). AS1411 aptamer (MW = 8486), B (**d**). AS1411-PEG-PDLLA (AS-PP), B (**e**). The physical mixture of AS1411 and HOOC-PEG-PDLLA without the addition of the coupling agent.

AS1411 aptamer, HOOC-PEG-PDLLA and AS-PP polymer were characterized by mass spectrometry (MS), ^1^H-NMR and polyacrylamide gel electroporesis (PAGE) respectively. The results were shown in Figure 3. Figure 3A (a) showed the molecular weight of NH_2_-AS1411 aptamer was 8486.0. Figure 3A (b) was the 1H-NMR spectrum of HOOC-PEG-PDLLA polymer material. PAGE was utilized to examine the conjugation of AS1411 to HOOC-PEG-PDLLA, and to demonstrate successful removal of unconjugated aptamers after the reaction. The mixing of aptamer and NPs without the addition of the coupling agent (AS+PP, Figure 3B (e)) did not show any band of conjugated aptamer after repetitive washing by ultracentrifugation (10000 × g, 15°C, 15 minutes) in centrifugal filter tubes (MWCO 10k), indicating a lack of non-specific interaction between the aptamer and HOOC-PEG-PDLLA. However, conjugation with the addition of EDC (AS-PP, Figure 3B (d)) leads to RNA bands consistent with RNA covalently bound to the HOOC-PEG-PDLLA even after the same repetitive washing by ultracentrifugation, which indicated AS1411-PEG-PDLLA polymer was obtained.

**Figure 4:**
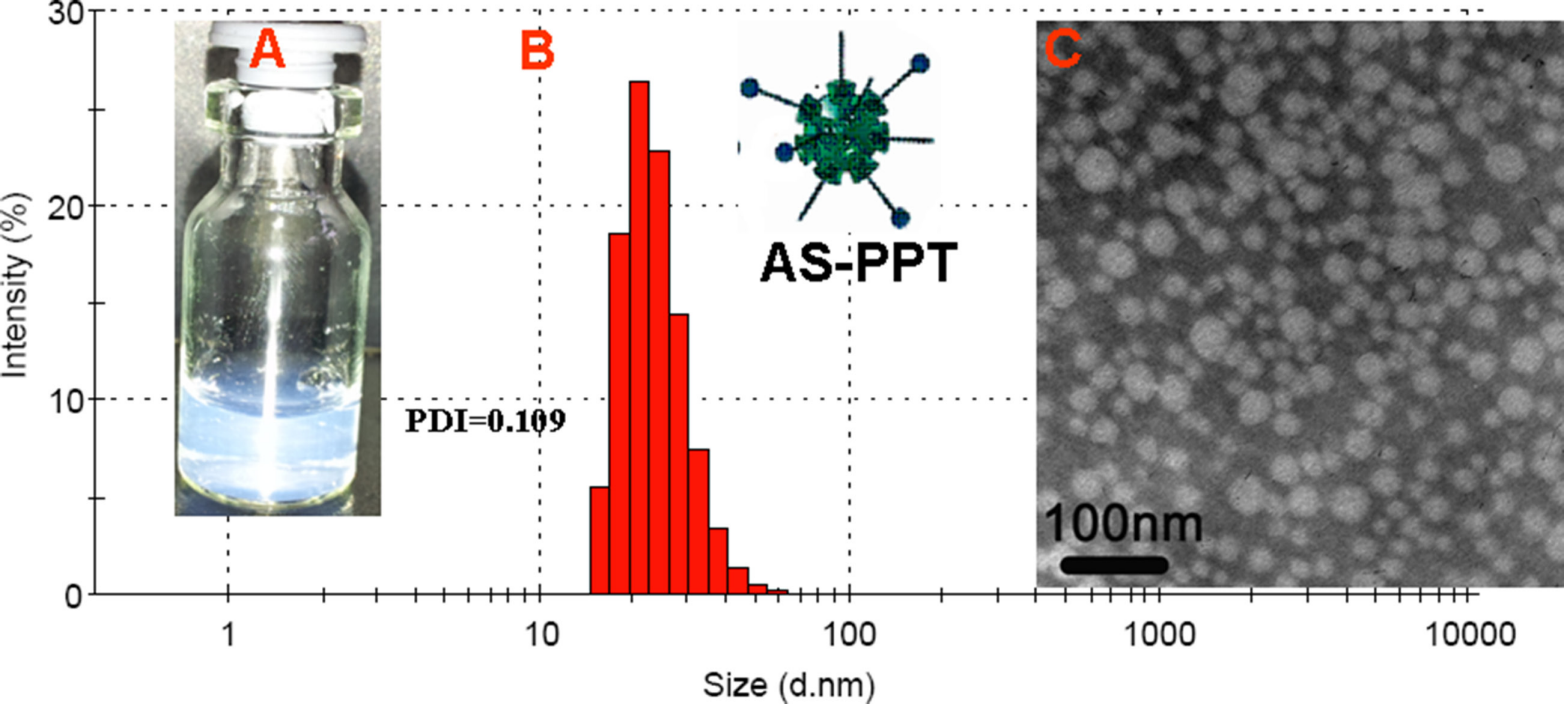
Preparation of AS-PPT (**A**) Morphology of AS-PPT. (**B**) Particle size. (*d* = 42 nm, PDI = 0.109. (**C**) TEM image of AS-PPT. Scale bars were 100 nm.

AS-PPT was prepared by solid dispersion technique using MPEG-PDLLA and AS-PP polymer (10:1). The appearance of AS-PPT was shown in Figure 4A, and the clear solution could be observed. DL and EE can be changed through varying the mass ratio of TP / carrier, and the highest DL and EE can reach to 6.1 ± 0.10% and 97.1 ± 1.01%, respectively. The AS-PPT (DL = 4%) was chosen to characterize in detail. The average size of AS-PPT was about 41.7 ± 1.5 nm (Figure 4B.) and PDI was 0.100 ± 0.023. Li Tang and his coworkers have proved that the 50 nm nanomedicine have highest anticancer effect for its highest tumor tissue retention, penetration and efficient cancer cell internalization [31]. This maybe one reason for its higher anticancer effect in the following studies. Figure 4C was the TEM image of AS-PPT in water, which showed AS-PPT were uniform and spherical shapes in solution, and such Figure was consisted with the result tested by Malvern Nano-ZS 90 laser particle size analyzer.

### The selectivity and antitumor activity of AS-PPT *in vitro*

**Figure 5:**
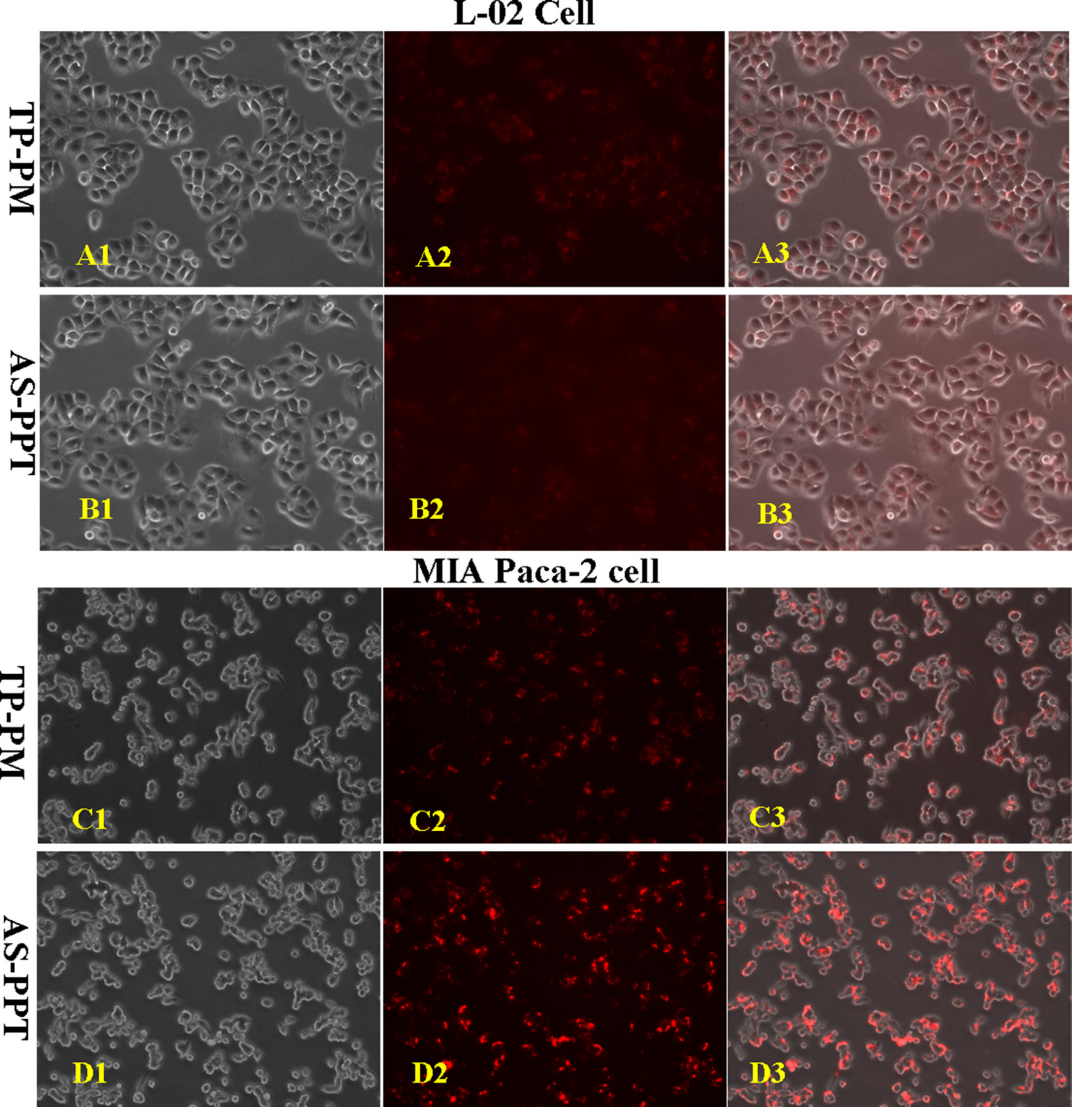
Cellular selectivity: The fluorescence intensity of cy3-labeled TP encapsulated by TP-PM and AS-PPT after incubation with human liver cell line L-02 and human pancreatic carcinoma cell line MIA PaCa-2 for 2 h, respectively (**A1, B1, C1, D1**) were the cell images observed with interference-phase contrast microscope. (**A2, B2, C2, D2**) were the cell images observed with fluorescence microscope. (**A3, B3, C3, D3**) were the aforementioned overlay images. (×200).

To confirm the specific binding ability of AS-PPT to cancerous cells, either the Cy3-labeled TP loaded in AS-PPT or Cy3-labeled TP loaded in TP-PM were incubated with human pancreatic carcinoma cell line MIA PaCa-2 and human liver cell line L-02 respectively. Figure 5 (A1–D1) and Figure 5 (A2–D2) were the cell images observed with interference-phase contrast microscope and fluorescence microscope, respectively, and Figure 5 (A3–D3) were their overlay images. Figure 5A2 showed the close fluorescence intensity with Figure 5B2, which illustrated AS-PPT had no specific-targeting L-02 cells compared with TP-PM, in addition, the Figure 5A3 and Figure 4B3 further confirmed such results. In contrast, MIA PaCa-2 cells in AS-PPT group (Figure 5D2) showed stronger fluorescence intensity than that of TP-PM group (Figure 5C2). Especially, Figure 5D3 showed stronger red fluorescence signal in MIA PaCa-2 cells than that of Figure 5C3, which implied AS-PPT group had higher internalizing ability with MIA PaCa-2 cell than that of TP-PM group. This should be attributed to the specific binding ability of AS1411 aptamer with nucleolin overexpressed on the surface of cancerous cells.

**Figure 6:**
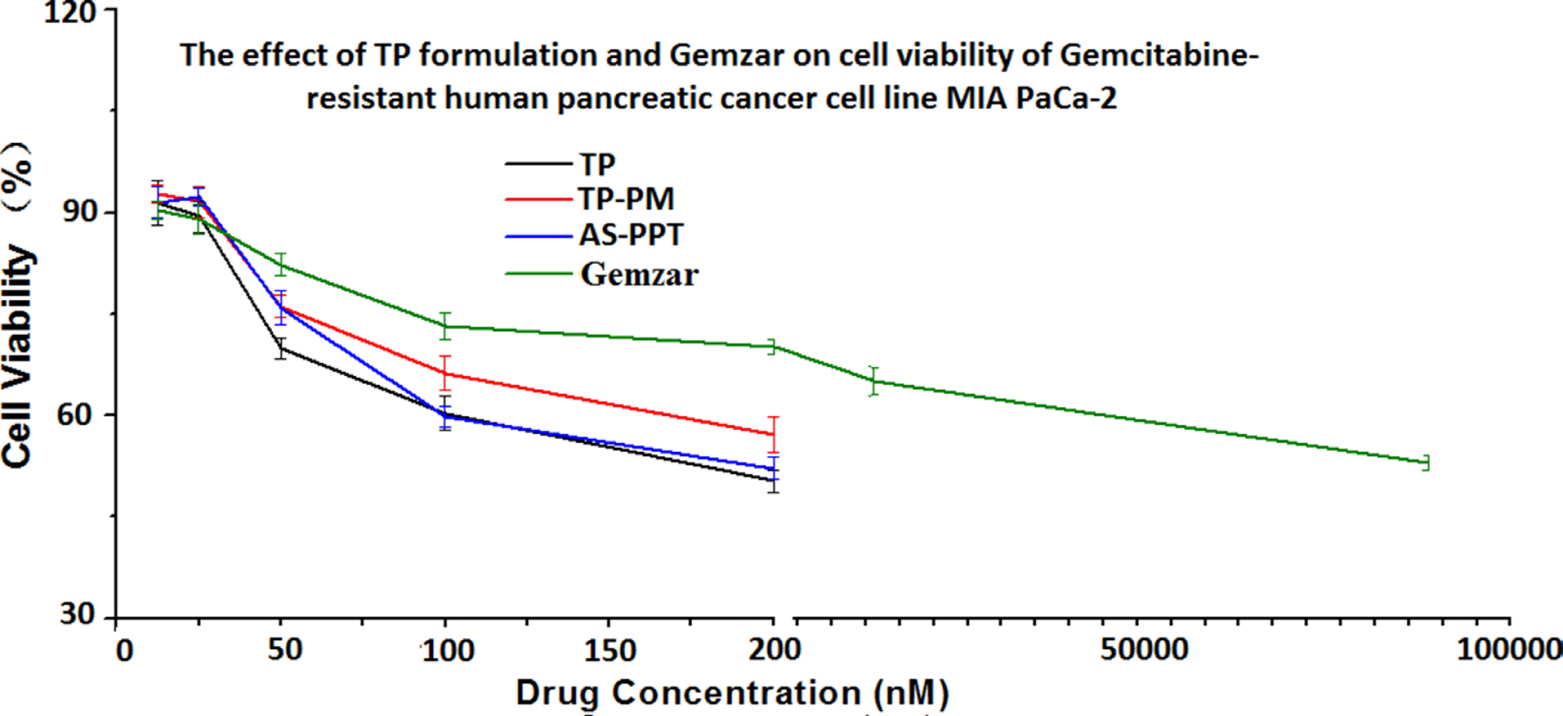
Cytotoxicity of different concentration of TP formulation and Gemzar^®^ on MIA PaCa-2 cell line after incubated for 48 h Error bars represent the standard deviation (*n* = 6).

Considering the controlled-release behavior of TP-PM and AS-PPT [26], we compared the cytotoxicity of them with free TP and Gemcitabine on MIA PaPa-2 cells for 48 h. The viability of cells was measured consecutively at arranged time points using MTT assays. The results showed that the presence of TP, TP-PM and AS-PPT significantly reduced MIA PaPa-2 cell viability in a dose-dependent manner (Figure 6). It seemed that AS-PPT and free TP have the same antitumor activities, but more activities than TP-PM *in vitro*, which might be ascribed to the mediation of AS1411 strengthening the cell endocytosis of AS-PPT. Compared with Gemcitabine, lower nanomolar concentrations of each TP formulation induced pancreatic cancer cell death, indicating TP exhibited more antitumor activities than Gemcitabine *in vitro*.

### *In vivo* distribution studies

**Figure 7:**
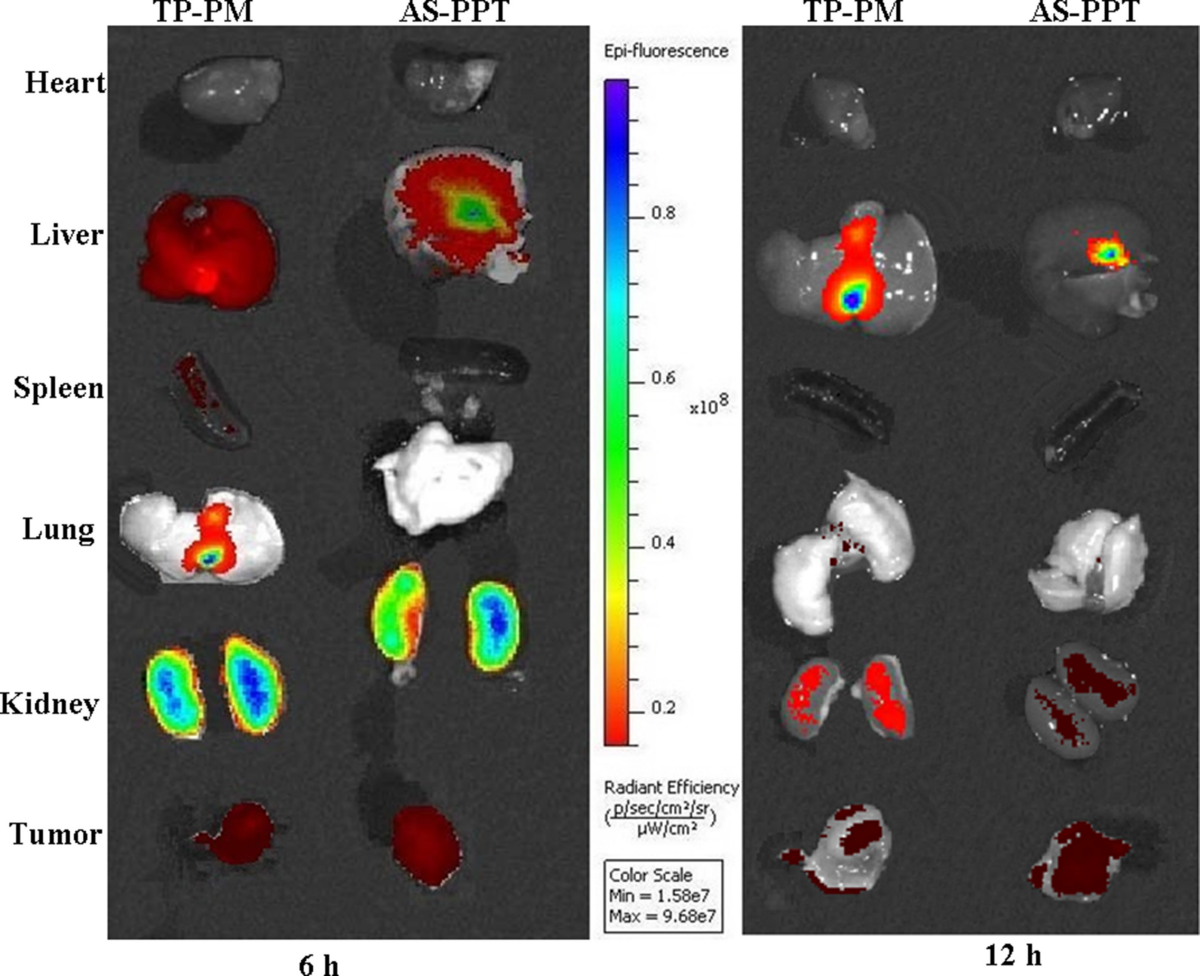
Tissue distribution: Localization of Cy3-labeled TP by biophotonic imaging-based analysis after administration of rats with TP-PM and AS-PPT at 6 h and 12 h time point The intensity of fluorescence signal in the isolated hearts, livers, spleens, lungs, kidneys and tumor were presented.

To evaluate whether the AS1411 aptamer-mediated nanocarrier loading TP can indeed specifically targeting tumors, we used biophotonic imaging technology to examine the tissue distribution of Cy3-labeled TP delivered by TP-PM and AS-PPT. Figure 7 showed that both formulations were prone to accumulate in liver and kidney for the initial 6 h, which should be attributed to the rapid uptake by mononuclear phagocyte system (MPS) and glomerular filtration [32, 33]. The tumor of two groups showed the same fluorescence signals intensity at 6 h, which should be ascribed to their nanostructure, this is also in line with the findings of Li Tang [31]. In contrast, the fluorescence signal intensity of AS-PPT in tumor were significantly higher than that of TP-PM at 12 h (Figure 7), which may be attribute to that AS1411 aptamer-mediated nanoparticle interacted highly with the cancerous cells and inhibited the efflux of AS-PPT from the cell by P-glycoprotein. In addition, other tissues such as spleen and lung of TP-PM group showed negligible fluorescence signal at 6 h post-injection. And the fluorescence signals in heart, spleen and lung were hardly detected in AS-PPT administration group at either 6 h or 12 h.

### *In vivo* pharmacodynamics of AS-PPT

**Figure 8:**
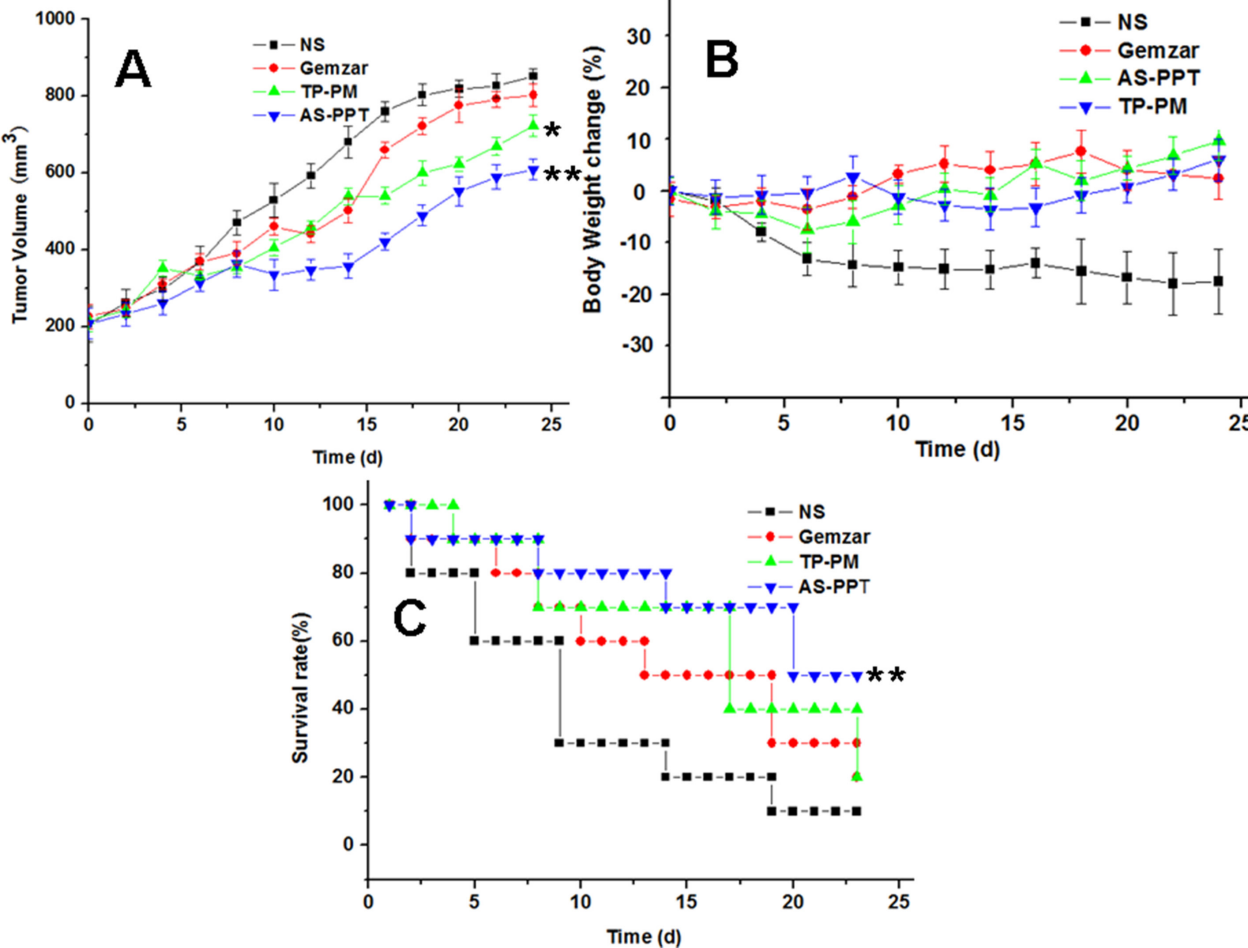
(**A**) Tumor volume changes of MIA PaCa-2 cell–xenografted BALB/c nude mice after treatment, significance were assessed compared with Gemzaer, *p < 0.05 (**B**) Body weight changes of BALB/c nude mice after treatment. (**C**) Survival rate of mice. (10 mice each group), significance were assessed compared with TP-PM, Gemzaer, *p < 0.05.

To evaluate the pharmacodynamics of AS-PPT, we compared the *in vivo* antitumor efficiency of TP-PM, AS-PPT and Gemzar^®^ in xenograft mouse model of MIA PaCa-2. When the treatment began, the body weight and tumor volume were monitored and calculated every two days. The results were shown in Figure 8. We found that TP-PM and AS-PPT can significantly inhibited tumor growth compared with negative control (NS) and positive control (Gemzar^®^), and the therapeutic group (AS-PPT) showed the slowest growth rate of tumor volume among the four groups. From the Figure 8A, we can see that TP-PM, AS-PPT and Gemzar ^®^ group showed similar growth rate of tumors during the early stage, furthermore, both the TP-PM and AS-PPT group showed slower tumor growth rate during the late stage of treatment, especially, that of AS-PPT group was the slowest among four groups, the reason might be the sustained release of TP from AS-PPT and higher AS-PPT accumulation in cancerous cells mediated by AS1411 aptamer. There were significant differences between positive group (Gemzar ^®^) and therapeutic groups, respectively (*P < 0.05). Weight profiles showed a largish loss within the first week in negative control group (NS), TP-PM and AS-PPT groups than that of positive control group (Gemzar^®^), whereas the body weight of the mice in three therapeutic groups (TP-PM, AS-PPT, Gemzar^®^) all exhibited steady increase than that of negative control group after the 10th day (Figure 8B). Contrary to our expectation, the body weight of positive control group (Gemzar^®^) exhibited higher increase than that of TP-PM and AS-PPT group, the reason should be the toxicity of TP released from delivery system, which bring the discomfort to mice.

Evaluation of the effect of AS-PPT on mice survival was accomplished and displayed in Figure 8C. It can be seen that more than half of mice in negative control group and positive control group died within 10, 20 days, respectively. Half of mice in TP-PM group died within 18 days, however, the mice with 40% survived for 24 days. In contrast, the therapeutic group administrated AS-PPT still remained half mice after 24 days. Significance were assessed compared with the control group of Gemzar ^®^ (*p < 0.05).

**Figure 9:**
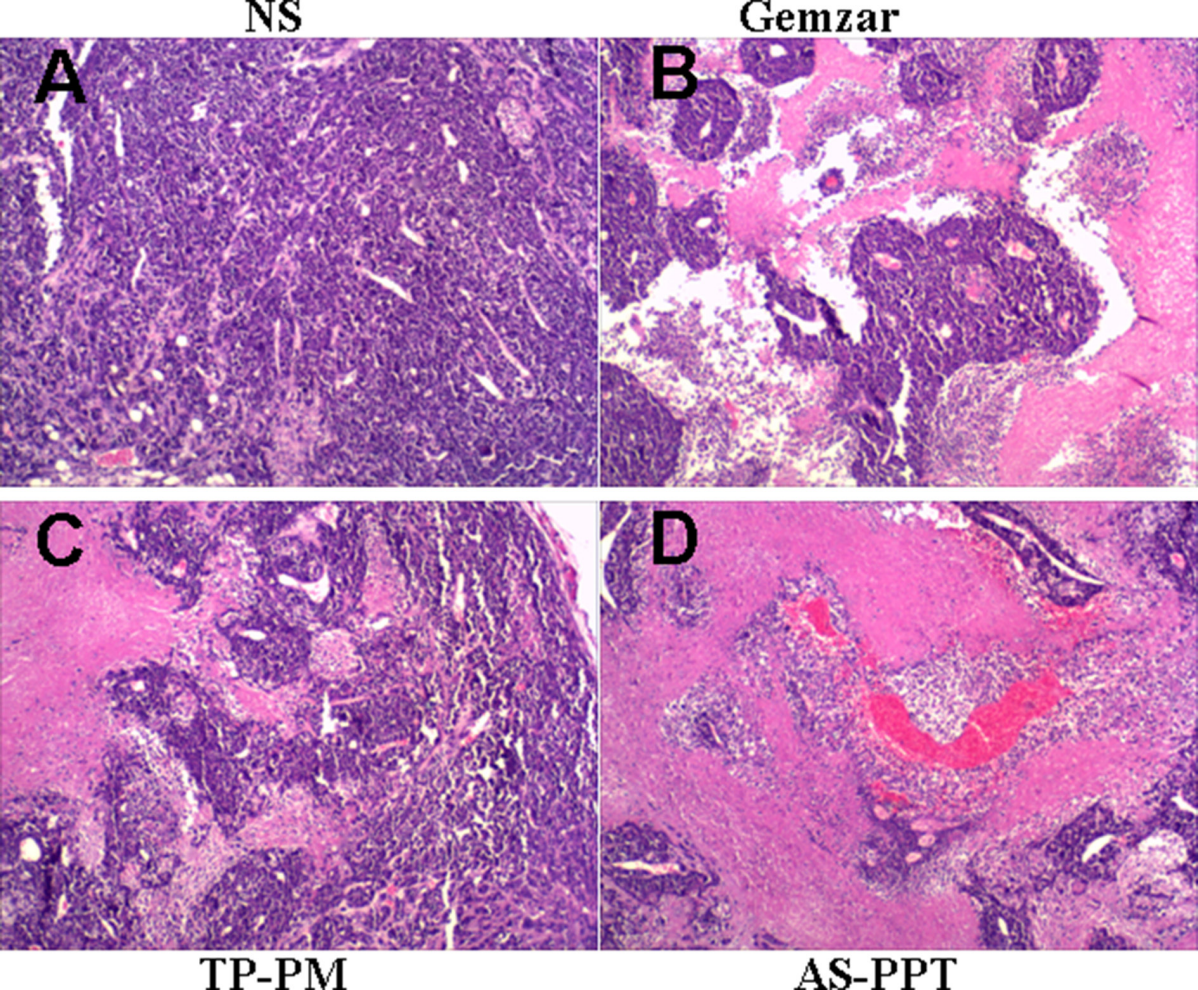
Light micrographs of tumor in mice treated with (**A**) NS, (**B**) Gemzar, (**C**) TP-PM, and (**D**) AS-PPT (×400): which were collected from mice after the last treatment and were stained haematoxylin and eosin.

Figure 9 (A, B, C, D). represents an optical micrograph of tumor tissue of mice treated with NS, Gemzar^®^, TP-PM and AS-PPT, respectivley. Many scattered focal cancer cell nests are in gland tube-like arrangement with red blood cell aggregation within the lumen, in addition, the tissue necrosis and inflammatory cell infiltration can be observed in Figure 9A. All these indicated the NS has no inhibition of cancer cells. Figure 9B shows a wide range of cancer cell necrosis and inflammatory cell infiltration, indicating Gemazar with good effect for inhibiting cancer cells. Figure 9C is the group with administration of TP-PM, from the figure, we can find a lot of necrotic tissue and inflammatory cell infiltration, indicating that TP-PM has good anti-gemcitabin-resistant pancreatic cancer efficacy, these should be ascribed to nanocarrier facilitating TP with multiple therapeutic target accumulating in tumor tissue. From the Figure 9D, a lot of cancer cells undergo apoptosis, scattered focal cancer cell nests arranged in gland tube-like. AS-PPT exhibited superior anticancer efficacy compared to Gemazar^®^ group and TP-PM group, which should be attributed to AS1411 aptamer provide AS-PPT an approach to achieve nucleolin-specific delivery and promoting endocytosis of cancerous cells for AS-PPT, which is also consistent with the survival results.

## CONCLUSION

Effective treatment of pancreatic cancer is a long cherished object for medical workers. A viable strategy have been developed, such as surgery, radiotherapy and chemotherapy, etc. However; each suffers its own specific limitations mainly arising from local recurrence and metastatic spread of such disease. Pathological site-specific delivery of active agents was considered to be a perfect strategy. Nanoscience generated nano-sized carriers provides potential to realize such purpose based on EPR effect, however, the sole nanocarrier with no specific ligands are not sufficient to deliver drugs to target sites. Nowadays, ligand-modified nanoscale preparations have been considered to effectively improve the anti-tumor efficacy based on EPR effect and subsequent endocytosis of cancerous cells via ligand–receptor interaction. As one of the most excellent targeting ligands, aptamers can selectively bind to a variety of targets ranging from small molecules to whole cells due to their three-dimensional structures even without knowledge of characteristic protein profiles on cell surface. Triptolide posses multiple therapeutic targets for cancer, however, hydrophobicity and toxicity due to non-specific exposure to healthy tissues hindered its clinical translation. Currently, we constructed AS1411 aptamer modified PEG-PDLLA micelle loading TP with an average diameter of 41.7 ± 1.5 nm. The *in vitro* results showed that AS-PPT had specific binding ability with human pancreatic carcinoma cell (MIA PaCa-2) but no specific-targeting L-02 cells compared with TP-PM. This should be attributed to the specific binding ability of AS1411 aptamer with nucleolin overexpressed on the surface of cancerous cells.

*In vivo* tumor inhibition study revealed that the AS-PPT has superior anti-tumour efficacy on PC cells when compared with TP-PM and Gemzar^®^. Furthermore, *in vivo* distribution studies showed AS-PPT kept more TP accumulation in tumor and extend tumor residence than TP-PM, this should be attribute to the specific interaction between AS1411 aptamer and nucleolin significantly enhanced cellular uptake of AS-PPT and inhibited the efflux of AS-PPT from the cell by P-glycoprotein. Based on the foregoing experimental data, we concluded that AS1411 aptamer-medicated nanocarrier can facilitate TP accumulating in tumor tissues via EPR effect, when they entered the tumor interstitial space, AS1411 aptamer presented on the AS-PPT surface quickly recognized cancerous cell followed by AS1411-nucleolin interaction, and consequently enhanced the inhibition of tumor growth. In conclude, AS-PPT have great potential for reversing the drug resistance of pancreatic cancer.

## MATERIALS AND METHODS

### Materials

Materials used included D,L-lactide (Aldrich, USA), monomethoxy poly(ethylene glycol) (MPEG_2000_, Aldrich, USA), stannous octoate (Sn(Oct)_2_). The carboxy terminated poly(ethylene glycol)–block–poly(d,l-lactide) (HOOC-PEG_2000_-PDLLA_6000_) was purchased from Xi'an ruixi Biological Technology Co., Ltd (China). Aptamer NH_2_-AS1411 (5′GGTGGTGGTGGTTGT GGTGGTGGTGG-3′-NH_2_) and Sulfo-Cyanine3-Triptolide (Cy3-TP) were purchased from Guangzhou RiboBio Co., Ltd (Guangzhou, China). Dulbecco's modified Eagle's medium (DMEM), 3-(4,5-dimethylthiazol-2-yl)-2,5-diphenyl tetrazolium bromide (MTT, Sigma, St. Louis, MO). Dehydrated alcohol, dimethylformamide (DMF), acetonitrile, dichloromethane (DCM) and petroleum ether were purchased from Huasheng Chemicals (Qingdao, China), dimethyl sulfoxide (DMSO, Huasheng Chemicals, Qingdao, China), Gemcitabine hydrochloride for injection (Gemzar^®^) was purchased from Eli Lilly Co, Ltd and triptolide (TP, Purity > 99%) was bought from Chengdu Biopurify Phytochemicals Co, Ltd (Chengdu, China). Gemcitabin-resistant human pancreatic cancer cell line (MIA PaCa-2) and human liver cell line L-02 were purchased from Shanghai Bioleaf Biotech Co., Ltd. BALB/c nude mice (8–10 weeks, 20 ± 2 g) were obtained from the Institute of Experimental Animals in Chinese Academy of Medical Science.

The animals were housed at temperature of 20–22°C, relative humidity of 50–60% and 12 h light-dark cycles. Free access to food and water was allowed. All animals would be in quarantine for a week before treatment. All animals care and experimental procedures were conducted according to Institutional Animal Care and Use guidelines.

### Synthesis and characterization of AS1411-PEG-PDLLA polymer

AS1411-PEG_2000_-PDLLA_6000_ polymer was synthesized by EDC/NHS technique [34, 35] Briefly, some HOOC-PEG_2000_-PDLLA_6000_ polymer suspended in 5 ml DNase/RNase-free water (10 μg/μL) were incubated with 200 μL EDC (400 mM) and 200 μL NHS (200 mM) for 30 min at room temperature with gentle shaking. The NHS-activated nanomicelle were reacted with 3′-amino-RNA aptamer (5′GGTGGTGGTGGTTGTGGTGGTGGTGG-3′-NH_2_, 1 μg/μL). The reaction was lasted for 2 h with constant mixing at room temperature. Then the resulted AS1411-PEG_2000_-PDLLA_6000_ polymer (AS-PP) were purified with DNase/RNase-free water (15 mL) by ultrafiltration (10000 × g, 15°C, 15 minutes) in centrifugal filter tubes (MWCO 10 k). The AS-PP polymer were resuspended in DNase/RNase-free water and then lyophilized to be dried powder for further use. The preparative process was shown in the Figure 2.

The AS-PP polymer was confirmed on 10% TBE-Urea PAGE. HOOC-PEG-PDLLA Nano-micelle were incubated as above without the crosslinker (−EDC) to confirm covalent conjugation. AS-PP (+EDC), AS+PP (−EDC) were separated by PAGE. The molecular weight (MW) DNA marker and free aptamer served as standards for 50 and 25 base pair band on the gel.

### Preparation and characterization of TP-PM and AS-PPT

### Preparation of TP-PM

The synthesis, preparation and characteristics of MPEG_2000_–PDLLA_6000_ diblock copolymers and TP-PM have been described in our previous publications [26, 35]. To prepare TP-PM, some MPEG-PDLLA copolymer and TP were dissolved in 3 mL of dehydrated acetonitrile under vigorous stirring. After all contents were dissolved, the solution was evaporated on a rotary evaporator under reduced pressure at 60°C. Upon acetonitrile evaporation, a homogenous coevaporation was obtained. TP was distributed into polymeric carriers in an amorphous form. Then, the coevaporation was dissolved in 5 ml of water at 65°C to create the TP polymeric micelle solution, and the solution was filtered with a 0.22 μm filter to obtain a clear solution. Then, the solution was lyophilized in a freeze dry system to obtain a dried powder.

### Preparation of AS-PPT

PEG_2000_-PDLLA_6000_ copolymer and AS-PP freeze-dried powder (at a ratio of 10:1), TP were dissolved in 500 μL of dehydrated acetonitrile under vigorous shaking. After all contents were dissolved, the solution was evaporated under reduced pressure at 60°C. Then, the coevaporation were dissolved in 1 ml of water at 60°C to create the AS-PPT solution, and the solution was filtered with a 0.22 μm filter to obtain a clear solution. Then, the solution was lyophilized in a freeze dry system to obtain a dried powder.

The drug loading (DL) and encapsulation efficiency (EE) of AS-PPT were determined as follows: First, a standard absorbance curve was established with a series of standard TP solution to facilitate the determination of the exact amount of drug within the TP-PM and AS-PPT (data not shown). Determination of the TP quantity within the AS-PPT was done by high-performance liquid chromatography (HPLC. The column temperature was 30°C. The mobile phase was composed of methanol/acetonitrile/water (15/20/65, v/v/v %). The flow rate was 1.0 mL/min and the detection wavelength is 218 nm. Based on this data, the DL and EE of TP-PM and AS-PPT were calculated according to eqn. (1) and (2):

$$\text{DL}=\frac{\text{TP}}{\text{Amount of the Powder}}\times 100\%$$ (1)

$$\text{EE}=\frac{\text{DL}}{\text{Amount of TP in feed}}\times 100\%$$ (2)

The particle size of prepared AS-PPT nano-micelle was determined using a Malvern Nano-ZS 90 laser particle size analyzer after equilibration for 10 min. The morphological characteristics of the AS-PPT were examined by transmission electron microscope (TEM, H-6009IV, Hitachi, Japan).

### The Selectivity and antitumor activity of AS-PPT *in vitro*

To confirm the specific binding ability of AS-PPT to cancer cells, the AS-PPT (Cy3-labeled TP) and the TP-PM (Cy3-labeled TP) were respectively incubated with human pancreatic carcinoma cell line MIA PaCa-2 and human liver cell line L-02. Briefly, cells were plated at a density of 1 × 10^4^ cells per well in 100 μL of DMEM medium in 96-well plates and grown for 24 h. The cells were then exposed to TP-PM and AS-PPT at the same concentrations of cy3-labeled TP for 2 h. After incubation, the cells were washed using PBS buffer solution for three times to remove the free TP, TP-PM and AS-PPT. Then the cells were observed with fluorescence microscope.

To confirm whether AS-PPT have more antitumor activity than free TP, TP-PM and Gemzar^®^, the MIA PaCa-2 cells were incubated with the different TP concentrations of free TP solution, TP-PM, AS-PPT and Gemzar^®^ for 48 h at 37°C. After that, the viability of cells was measured consecutively at arranged time points using 3-(4,5-dimethylthiazole-2-yl)-2,5-di-phenyl tetrazolium bromide (MTT, Sigma-Aldrich) assays.

All the results obtained from MTT assays were confirmed by repeating at six individual experiments, and all data were expressed as the mean ± S.D.

### The tissue distribution and pharmacodynamics of AS-PPT in a xenograft mouse model of CPC

### Tissue distribution

For visually showing the tissue distribution of AS-PPT in animal model of CPC, fluorescence imaging will be performed using an IVIS^®^ 200 imaging system. Eight eight-week-old healthy female BALB/c nude mice (18–20 g body weight) will be inoculated with 100 μl of MIA PaCa-2 cell (1 × 10^7^/ml) suspended in normal saline on the right flank. Tumor size will be measured every other day using vernier calipers in two dimensions. Individual tumor volumes (V) were calculated using the formula V = [length × (width)^2^] M/2, where length is the longest diameter and width is the shortest diameter perpendicular to length. Once the tumors reached 200 mm^3^, the mice will be randomly divided into 2 groups (*n* = 4 for each group), and respectively subjected to TP-PM and AS-PPT at the same dose of 0.2 mg/kg of cy3-labeled TP via tail vein injection. Two mice of each group will be sacrificed at 6 h and 12 h after the administration and the major organs (heart, liver, spleen, lung, kidney and tumor) will be collected. The fluorescence signal of those organs in each group will be detected using Xenogen IVIS imaging system (Xenogen Imaging Technologies, Alameda, CA). Excitation (λ_ex_ = 445–490 nm) and Emission (λ_em_ = 515–575 nm) filters were used. Identical illumination settings including exposure time (5 s), binning factor (4), f-stop (2) and fields of view (15 cm for width and length, respectively), will be used for all image acquisition. Fluorescent and photographic images will be acquired and overlaid. The *in vivo* experiments were performed in accordance with institutional guidelines and in compliance with national and international law and policies. All efforts were made to minimize the number of animals used and their suffering.

### Pharmacodynamics

Briefly, fifty two eight-week-old healthy female BALB/c nude mice (18–20 g body weight) were injected subcutaneously on the right back with 0.1 mL of cell suspension containing MIA PaCa-2 cell (1 × 10^7^/ml). Treatment was initiated when the tumor reached a size of approximately 200 mm^3^, the mice were randomly divided into four groups containing thirteen animals and samples were administered via vein once a week for three weeks. Group 1 kept as the negative control group and receive 0.1 mL dose of normal saline (NS), group 2 received 0.1 mL of Gemzar^®^ with 20 mg/kg (as the positive control group), group 3 received 0.1 mL of TP-PM with dosage of 0.32 mg/kg TP and group 4 received 0.1 mL of AS-PPT with dosage of 0.32 mg/kg TP. During this period, the mice were observed continuously for relevant indexes, such as body weight, tumor volume and survival rate. Three mice of each group were sacrificed on the 3th week post-treatment and the tumor were harvested and fixed in 10% buffered formaldehyde solution and then embedded in paraffin. Sections of 3–5 mm were stained with hematoxylin and eosin (H & E) and observed on light microscope and electron microscopic measurements.
